# Acute *Toxoplasma gondii* Infection among Family Members in the United States

**DOI:** 10.3201/eid1912.121892

**Published:** 2013-12

**Authors:** Despina G. Contopoulos-Ioannidis, Yvonne Maldonado, Jose G. Montoya

**Affiliations:** Stanford University School of Medicine, Stanford, California, USA (D.G. Contopoulos-Ioannidis, Y. Maldonado, J.G. Montoya);; Palo Alto Medical Foundation Toxoplasma Serology Laboratory, Palo Alto, California, USA (D.G. Contopoulos-Ioannidis, J.G. Montoya)

**Keywords:** acute Toxoplasma infection, United States, families, Toxoplasma, toxoplasmosis, Toxoplasma gondii, parasites, protozoa, acute toxoplasmosis

## Abstract

We investigated 32 families of persons with acute toxoplasmosis in which >1 other family member was tested for *Toxoplasma gondii* infection; 18 (56%) families had >1 additional family member with acute infection. Family members of persons with acute toxoplasmosis should be screened for infection, especially pregnant women and immunocompromised persons.

Only isolated case reports and small case series have been published on acute *Toxoplasma* *gondii*. infections among family members (*1*–*6*). When a case of acute toxoplasmosis is identified in a family, additional household members might have been infected around the same time period; family members frequently share common exposures to food or environmental sources potentially contaminated with *T. gondii*. Identification of additional infections could lead to earlier implementation of appropriate interventions for persons in certain high-risk groups, such as immunocompromised persons and pregnant women.

Large-scale evaluation of the prevalence of acute toxoplasmosis among family members in the United States has not been performed (*4*). Therefore, we investigated the prevalence of acute toxoplasmosis among household and family members of patients who had acute toxoplasmosis.

## The Study

We performed a retrospective cohort study using data collected by the Palo Alto Medical Foundation Toxoplasma Serology Laboratory (PAMF-TSL; www.pamf.org), Palo Alto, California, USA, during 1991–2010. Patient blood samples were sent from diverse laboratories from throughout the United States, and testing was conducted at the PAMF-TSL. The study was approved by the Institutional Research Board at the PAMF Research Institute.

From the PAMF-TSL database, we identified families that 1) had an index case-patient with a diagnosis of acute toxoplasmosis and 2) had >1 additional household/family member who had been tested for *T. gondii* infection at PAMF-TSL. Details of the process used to identify additional household/family members are described in the Technical Appendix. All identified family/household members were categorized as acutely infected (<6 months before sample collection time); recently infected (6–12 months before sample collection time); chronically infected (>12 months before sample collection time); or never infected. The criteria used for this categorization are described in the Technical Appendix. These criteria are routinely used in the daily clinical practice at PAMF-TSL to estimate the most likely time of the *T. gondii* infection; the accuracy of these criteria has been previously validated (*7*–*11*).

All identified families were categorized in 3 family groups (Technical Appendix). Group 1 consisted of families with an index case-patient who had acute toxoplasmosis and >1 additionally tested family/household member who had acute or recently acquired *T. gondii* infection. Group 2 consisted of families with an index case-patient who had acute toxoplasmosis; >1 additionally tested family/household member who had chronic *T. gondii* infection; and no other tested household members who had evidence of acute or recently acquired *T. gondii* infection. Group 3 consisted of families with an index case-patient who had acute toxoplasmosis and in which no additionally tested family/household members showed evidence of *T. gondii* infection. 

We defined as prevalence of acute *T.* *gondii* infection in >1 family members (prevalence of group 1 families) the number of group 1 families divided by the total number of study families over the 20-year study period (primary endpoint). As secondary endpoint, we also calculated the prevalence of group 2 families. We also tested whether the IgG-Dye test titers and IgM-ELISA titers of the index case-patients were different across the 3 family groups by using the Kruskal-Wallis test. All analyses were done in Stata/SE version 12 (StataCorp LP, College Station, TX, USA).

Among 97,279 persons serologically tested for *T. gondii* in the PAMF-TSL over the 20 year study period, we identified 107 persons who had >1 person from their household with a diagnosis of acute toxoplasmosis and >1 additional household member serologically tested for *T. gondii* infection. Those 107 persons were grouped into 32 study families (Figure). Patient demographic and clinical characteristics are shown in Table 1; serologic test results for members of group 1 families are shown in Table 2, Appendix, and for members of groups 2 and 3 families in the Technical Appendix. 

**Figure:**
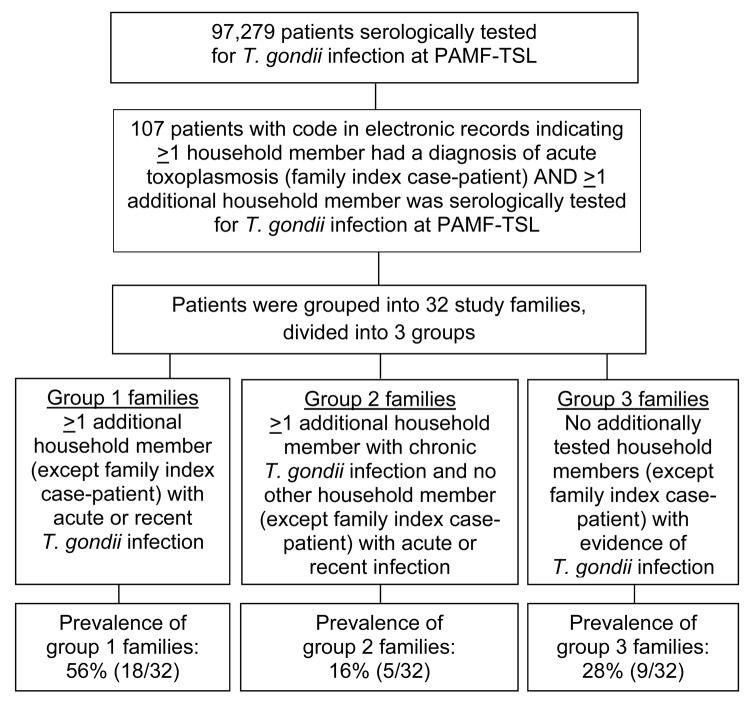
Flowchart for the identification of families with an index case-patient who had acute toxoplasmosis and >1 family member with acute or recent *Toxoplasma* *gondii*. infection. Data were extracted from the database of the Palo Alto Medical Foundation Toxoplasma Serology Laboratory (PAMF-TSL; Palo Alto, CA, USA), from patient samples sent to PAMF-TSL during 1991–2010 from laboratories throughout the United States.

**Table 1:** Demographic and clinical information for persons in the 18 group 1 study families identified from data on acute toxoplasmosis cases collected during 1991–2010 by the Palo Alto Medical Foundation Toxoplasma Serology Laboratory, Palo Alto, California, USA*

IC patient no.	Clinical information for IC	No. additional household members tested	Infection status of additional household members	Clinical information for additional household members	Risk factors reported by ≥1 household member
IC-1	LN	2	Wife: acute infection	Pregnant, first trimester	Ate raw lamb
			Daughter: no infection	NA	
			(Baby girl: status not ascertained)		
IC-2	8 wks pregnant	1	Husband: acute infection	LN	NR
			(Fetus: AF PCR–)		
IC-3	8 wks pregnant	1	Husband: acute infection	Asymptomatic	Contact with cat feces, eating undercooked meat, gardening
			(Baby boy: could not R/O CT; no follow-up beyond 1 mo of age)		
IC-4	27 wks pregnant	2	Husband: acute infection	NA	NR
			Son: acute infection	NA	
			(Fetus: AF PCR–)		
IC-5	11 wks pregnant	1	Husband: acute infection	NA	None
			(Fetus: AF PCR–)		
IC-6	Infant with CT	2	(Mother: acute infection)	NA	NR
			Father: acute infection	NA	
			Brother: acute infection	NA	
IC-7	LN, fever, headache	3	Wife: acute infection	LN	Poor cleaning of cooking surfaces
			Daughter 1: acute infection	Posterior cervical LN	
			Household member: chronic infection	NA	
			Son/daughter 2: not tested		
IC-8	13 wks pregnant	1	Husband: acute infection	NA	Ate deer meat that had positive results for *T. gondii* by PCR
			(Baby Boys A and B: status not ascertained)		
IC-9	22 wks pregnant	1	Husband: acute infection	NA	NR
			(Fetus: NA)		
IC-10	Pregnant, third trimester	2	Daughter 1: Recent infection	Asymptomatic	Children played in uncovered sandbox
			Daughter 2: acute infection	Asymptomatic	
			(Baby girl A: asymptomatic; CSF PCR–, could not R/O CT; baby girl-B: CT, macular scar, ascites, AF PCR+, CSF PCR+)		
IC-11	Infant with CT†	2	(Mother: recent infection)	NA	NR
			Father: recent infection	NA	
			Sister: no infection	NA	
IC-12	LN, fever, hepatitis	3	Wife: acute infection	LN	Ate raw lamb
			Household member 1: acute infection	LN	
			Household member 2: acute infection	NA	
IC-13	21 wks pregnant	1	Husband: acute infection	LN	Ate venison tartare
			(Fetus: CT, ascites, hydrocephalus; abortion)		
IC-14	Infant with CT	1	(Mother: acute infection)	NA	Ate bear meat; ate deer meat that had positive results for *T. gondii* by PCR
			Father: acute infection	Fever, flu-like symptoms	
IC-15	9 wks pregnant	1	Husband: acute infection	NA	None
			(Baby boy: status not ascertained)		
IC-16	Febrile illness (fibromyalgia)‡	3	Daughter 1: Recent infection	NA	Ate deer meat that had positive results for *T. gondii* by PCR
			Daughter 2: no infection	NA	
			Grandson: no infection	NA	
IC-17	Eye disease	3	Son: acute infection	NA	NR
			Daughter 1: acute infection	Asymptomatic	
			Daughter 2: no infection	NA	
IC-18	LN	1	Wife: Recent infection	NA	NR

*Mother-infant pairs were counted as 1 unit/household member; infection status of these is shown in parenthesis. IC, index case-patient; LN, lymphadenopathy; NA, not available; NR, not reported; AF, amniotic fluid; R/O, rule out; CT, congenital toxoplasmosis; CSF, cerebrospinal fluid. †Infant with CT with hydrocephalus, high bilirubin, abnormal liver function tests, low platelets, and positive PCR results on CSF. ‡Female patient taking chronic corticosteroids; patient died.

**Table 2:** Serologic test results for family index case-patients and additionally tested household members in the 18 group 1 study families*

Index case-patients (clinical information) and additional household members tested	IgG by dye test	ELISA results			AC/HS pattern	Avidity	Interpretation of infection type
		IgM	IgA	IgE			
IC-1 (LN)	512	8.3	8.5	3.1	Acute	ND	Acute
Wife†	4,096	3.2	10.3	1.1	Acute	ND	Acute
Daughter	<16	0.5	0.2	0	Nonreactive	ND	None
Baby girl	2,048	0 (ISAGA)	2	0.2	ND	ND	Status NA
IC-2 (8 wks pregnant)	8,000	5.7	13.9	2.6	Acute	ND	Acute
Husband	16,000	4.1	3.2	3.2	Acute	ND	Acute
Fetus	ND	ND	ND	ND	ND	ND	Status NA
IC-3 (8 wks pregnant)	16,000	4.6	3.4	1.1	Acute	Low (7.8)	Acute
Husband	8,000	7.3	>11	2.4	Acute	Low (4.4)	Acute
Baby boy	2,048	0 (ISAGA)	0	ND	ND	ND	Status NA
IC-4 (27 wks pregnant)	512	5.3	5.6	0.4	Equivocal	Low (2.8)	Acute
Husband	1,024	5.9	12.4	Negative	Acute	Low (5.4)	Acute
Son	16,000	7.2	>24	4.2	Acute	Low (10.5)	Acute
Fetus	ND	ND	ND	ND	ND	ND	Status NA
IC-5 (11 wks pregnant)	2,048	5.8	2	0.2	Acute	Low (6.7)	Acute
Husband	1,024	5.8	2.3	0	Acute	Low (13.2)	Acute
Fetus	ND	ND	ND	ND	ND	ND	AF PCR–
IC-6 (infant with CT)	32,000	12 (ISAGA)	>24	9.5	ND	ND	Congenital
Mother	32,000	10.5	>24	4.6	Acute	ND	Acute
Father	8,000	4.9	11	1.1	Acute	ND	Acute
Brother	2,048	5.4	5.1	0.2	Acute	ND	Acute
IC-7 (LN, fever, headache)	8,000	9.9	>11.2	>20	Acute	Low (1.3)	Acute
Wife	32,000	5.2	9.4	1.3	Acute	Low (1.0)	Acute
Daughter 1	1,024	>10.0	7.2	5.3	Acute	Low (1.2)	Acute
Household member	512	0.9	ND	ND	ND	ND	Chronic
IC-8 (13 wks pregnant)	512	7.9	5.7	ND	Acute	Low (7.4)	Acute
Husband	4,096	7.2	1.8	1.9	Acute	Low (11.0)	Acute
Baby boy A	1,024	0 (ISAGA)	0.2	ND	ND	ND	Status NA
Baby boy B	1,024	0 (ISAGA)	0	ND	ND	ND	Status NA
IC-9 (22 wks pregnant)	2,048	7.8	1.3	0.8	Acute	Low (1.8)	Acute
Husband	4,096	9.8	6.4	3.9	Acute	Low (6.6)	Acute
IC-10 (pregnant, third trimester)	4,096	5.4	9.4	2.9	Acute	Low (5.9)	Acute
Baby girl A	8,000	0 (ISAGA)	0.9	0.8	ND	ND	Status NA
Baby girl B	8,000	7 (ISAGA)	1.6	0.3	ND	ND	Congenital
Daughter 1	8,000	0.4	0.7	1.2	Equivocal	Low (12.5)	Recent
Daughter 2	8,000	0.7	>11.2	1.5	Acute	Low (15.9)	Acute
IC-11 (infant with CT)	8,000	12 (ISAGA)	4.4	ND	ND	ND	Congenital
Mother	8,000	2.7	ND	ND	Acute	ND	Recent
Father	8,000	0	0.4	0.8	Acute	Low (16.2)	Recent
Sister	<16	0	ND	ND	ND	ND	None
IC-12 (LN)	4,096	11.2	11.4	14.1	Acute	ND	Acute
Wife	8,000	>10.0	11.2	>14.0	Acute	Low (3.8)	Acute
Household member 1	8,000	>10.0	>20.0	>14.0	Acute	Low (2.4)	Acute
Household member 2	1,024	>10.0	10.2	14.9	Acute	Low (11.5)	Acute
IC-13 (21 wks pregnant; abortion)	1,024	8.3	0.7	ND	Acute	ND	Acute
Husband	4,096	8.6	6.5	ND	Acute	ND	Acute
IC-14 (infant with CT)	32,000	7 (ISAGA)	>11.2	ND	ND	ND	Congenital
Mother	8,000	5.6	>11.2	4.4	ND	Low (15.7)	Acute
Father	8,000	3.7	3.8	1.4	Acute	Low (16.3)	Acute
IC-15 (9 wks pregnant)	2,048	6.6	1.7	3.1	Equivocal	Low (4.3)	Acute
Husband	128	5.2	0.4	0.8	Equivocal	Low (8.0)	Acute
Baby boy	256	0	0	ND	ND	ND	Status NA
IC-16 (fibromyalgia; taking steroids; fever; patient died)	8,000	9.4	4.5	11	Acute	Low (0.7)	Acute
Daughter 1	2,048	3.2	3.8	ND	Equivocal	Low (4.6)	Recent
Grandson	<16	0	ND	ND	ND	ND	None
Daughter 2	<16	0	ND	ND	ND	ND	None
IC-17 (eye disease)	2,048	8.1	3.4	10	Acute	Low (6.8)	Acute
Son	32,000	9.8	ND	ND	ND	ND	Acute
Daughter 1	128,000	8	ND	ND	ND	ND	Acute
Daughter 2	<16	0	ND	ND	ND	ND	None
IC-18 (LN)	2,048	8.8	3.2	7.1	Acute	ND	Acute
Wife	1,024	2.6	1.2	0.4	Acute	ND	Recent

*Mother-infant pairs were counted as 1 unit/household member. Interpretation of results: IgG dye test, positive >16, negative <16; IgM ELISA, positive >2.0, equivocal 1.7–1.9, negative <1.6; IgM ISAGA (for infants <6 mo of age), positive 3–12, negative 0–2; IgA ELISA, positive >2.1, equivocal 1.5–2.0, negative ≤1.4; IgE ELISA, positive >1.9, equivocal 1.5–1.8, negative <1.4; avidity, low <20, equivocal 20–30, high >30. The categorization of AC/HS test results into acute, equivocal, and nonreactive is available at www.pamf.org/serology/images/achs_grid.html. AC/HS, differential agglutination; IC, index case-patient; LN, lymphadenopathy; ND, not done; ISAGA, immunosorbent agglutination assay; NA, not ascertained; AF, amniotic fluid; CT, congenital toxoplasmosis. Serologic test results, despite equivocal AC/HS, were consistent with acute infection in IC4 and IC15 and recent infection in daughter 1 of IC10 and IC16. †Pregnant woman who was serologically tested for toxoplasmosis because of her husband’s toxoplasmic lymphadenitis.

The prevalence of group 1 families in our study was 56% (18/32); group 2 families, 16% (5/32); and group 3 families, 28% (9/32) (Figure). The IgG-Dye test and the IgM-ELISA titers of the index case-patients were not significantly different across the 3 family groups (p = 0.27 for IgG and p = 0.07 for IgM) (Table 2, Appendix; Technical Appendix). For group 1 families, all additional family members with acute/recently acquired infection had serologic profiles (titers of IgG, IgM, and/or IgA/IgE and avidity) that were similar to those of the index case-patients, indicating that they were infected at about the same time (Table 2, Appendix).

## Conclusions

Our data provide preliminary evidence that multiple cases of acute *T.* *gondii* infection may occur among family/household members. These findings are particularly critical for persons at high risk from *T. gondii* infection, such as women who are or may become pregnant or immunocompromised persons. Interpretation of our study findings would have been clearer had the background prevalence of acute toxoplasmosis in the United States been known. Although no such population-level empirical data exist, we have identified at PAMF-TSL 889 patients with acute *T.* *gondii* infection over the 20-year study period (estimated prevalence ≈9/1,000 patients screened at PAMF-TSL; unpub. data). 

A limitation of our study is that the families tested at PAMF-TSL over this study period might represent a group in whom the prevalence of acute *T.* *gondii* infection in >1 family member has been overestimated. Only 4% of persons who had acute toxoplasmosis diagnosed at PAMF-TSL during the 20-year study period had samples sent from additional household members for *T. gondii* testing (32 index case-patients with acute toxoplasmosis/889 acute infections). The collection of those additional samples depended solely on the response of the referring physicians to a 1-time written request for testing of additional family members. It is possible that the response of the primary care providers to this request would have been more likely if any of those additional family/household members had symptoms suggestive of acute toxoplasmosis. In addition, the IgG-Dye test and IgM-ELISA titers of the index case-patients did not predict which families would have additional household members with acute toxoplasmosis.

Further replication of the estimated prevalence of acute *T.* *gondii* infection in consecutive US families is needed. Future studies might also compare the *T. gondii* serotypes among index case-patients and family members (type II vs. non–type II) (*12*), which could help clarify whether certain serotypes are more likely to be associated with family outbreaks. Moreover, it would be useful to screen for antibodies to sporozoite-specific antigens (*13*), which can provide further insight regarding the source of *T. gondii* infection that is more likely to be associated with acute toxoplasmosis in >1 family member (e.g., sporozoite-specific, related to contact with cat feces, vs. bradyzoite-specific, related to ingestion of undercooked meat [*14*]).

When a case of acute toxoplasmosis is diagnosed, screening of additional family members should be considered, especially if pregnant women or immunocompromised patients live in those households, so that appropriate preventive strategies and/or therapeutic interventions are applied. These within-family clusters of cases are not easy to predict based solely on clinical or epidemiologic information, except for situations of sharing common meal (i.e., with undercooked meat), because it is unlikely that other risk factors would be different. Thus, only routine serologic screening of household members of acutely infected persons might identify such acute *T.* *gondii* infections.

Technical Appendix Supplementary methods and results from study of families of persons with acute toxoplasmosis using data collected in the Palo Alto Medical Foundation Toxoplasma Serology Laboratory, Palo Alto, California, USA, from patient samples sent to PAMF-TSL during 1991–2010 from laboratories throughout the United States.
