# Supplementary material for: Acute Toxoplasma gondii Infection among Family Members in the United States
Source: Emerg Infect Dis. 2013 Dec;19(12):1981–4. doi: 10.3201/eid1912.121892 (PMC3840881; doi:10.3201/eid1912.121892)
Supplement: Technical Appendix — Supplementary methods and results from study of families of persons with acute toxoplasmosis using data collected in the Palo Alto Medical Foundation Toxoplasma Serology Laboratory, Palo Alto, California, USA, from patient samples sent to PAMF-TSL during 1991–2010 from laboratories throughout the United States. [file 12-1892-Techapp-s1.pdf]

Article DOI: http://dx.doi.org/10.3201/eid1912.121892

Acute *Toxoplasma gondii* Infection among Family Members in the United States

Technical Appendix

Supplementary Methods

Study Design

We performed a retrospective cohort study using data collected in the Palo Alto Medical Foundation Toxoplasma Serology Laboratory (PAMF-TSL) (*1*) from 1/1/1991 to 12/31/2010 to identify families with a family/household member diagnosed with acute toxoplasmosis (index case [IC]) and at least one additional family/household member tested for *Toxoplasma gondii* infection. At PAMF-TSL, whenever a case of acute toxoplasma infection was serologically diagnosed, the PAMF-TSL medical consultant routinely requested screening of additional family/household members for *T. gondii* infection. This request was made in the written consult report to the primary care provider. This additional screening was offered free of charge.

Screening of PAMF-TSL Database (Code 90)

For each patient who was diagnosed at PAMF-TSL with acute toxoplasmosis (IC) AND who had at least one additional family/household member screened at PAMF-TSL for *T. gondii*, a specific code (code 90) was assigned in the electronic record of all these individuals (to the IC and their additional family/household members) indicating that they were part of a family/household tested for *T. gondii* infection. The criteria for the diagnosis of acute toxoplasmosis are described in the Diagnostic criteria section below. The code 90 was assigned even if only one and not all-additional family/household member was tested.

Index Cases

We screened the PAMF-TSL database for all individuals who were assigned the above code. For each particular family, the family/household member who was first tested at PAMF-TSL and diagnosed with acute toxoplasma infection was considered to be the IC for this particular family/household.

Identification of Additional Family/Household Members

To identify all the members from the same households we searched in the PAMF-TSL electronic database the individuals with similar last names and collection dates and hand reviewed the hard copies of their PAMF-TSL records. Individuals with the above code were then grouped into study families. If the IC was a congenitally infected infant or a pregnant woman, an additional household member, beyond the mother-infant pair, had to be tested as well, in order for this group of individuals to qualify for a household.

Serologic Tests

The serologic tests performed included the following: the Sabin-Feldman Dye test (IgG) (Laboratory Developed test) (*2*), IgM ELISA (Laboratory Developed test) (*3*) and immunosorbent IgM agglutination assay (IgM ISAGA) for infants less than 6 months of age (Laboratory Developed test) (*4*), immunoglobulin A (IgA) ELISA (Laboratory Developed test) (*5*), immunoglobulin E (IgE) ELISA (Laboratory Developed test) (*6*), the differential agglutination (AC/HS) test (Laboratory Developed test) [*7*] and the IgG avidity test (VIDAS Toxo-IgG Avidity kit; bioMérieux, Marcy-l'Etoile, France) [*8*].

Diagnostic Criteria

Acute *T. gondii* Infection within ≤6 Months from Sample Collection

We used the following three very strict composite criteria for the diagnosis of acute toxoplasma infection as reported elsewhere (*8–11*); A) IgG-Dye test titer ≥1:1024 AND IgM ELISA ≥5.0, AND acute pattern in the differential agglutination test; B) IgG-Dye test titer ≥1:1024 AND IgM ELISA ≥3.0 AND acute pattern in the differential agglutination test AND either IgA ELISA ≥5.0 or a low IgG avidity (< 10); C) IgG-Dye test titer ≤ 1: 512 AND IgM ELISA ≥5.0 AND acute pattern in the differential agglutination test AND either IgA ELISA ≥5.0 or low IgG avidity (< 10) (*8*,*10*). Patients meeting any of the above three composite criteria are likely to be infected within less than <6 months from the time of serum sampling. The above criteria were not applied if the IC was a congenitally infected infant or a patient with chorioretinitis. For congenitally infected infants there are special considerations: *first,* the diagnosis is based not only on serologic test results but also on the presence of clinical findings suggestive of congenital toxoplasmosis; *second,* IgM ISAGA is used instead of IgM ELISA (for infants < 6 months of age) and *third,* in 25-50% of infants with congenital toxoplasmosis the

IgM ISAGA can be negative at birth (*12*). Also, for patients with chorioretinitis, if the ocular findings are typical of toxoplasmosis, a high IgG titer, even in the absence of other serologic markers, can suggest a recent postnatally acquired *T. gondii* infection.

Recent Infection: within 6–12 Months from Sample Collection

High IgG-Dye test titer ≥1024; with or without a positive IgM-ELISA (<3.0) AND an acute pattern on the differential agglutination test.

Chronic *T. gondii* Infection Acquired in the Distant Past >12 Months from Sample Collection

IgG-Dye test titers < 1:1024 AND IgM ELISA < 3.0 AND differential agglutination test (if performed) with chronic (non-acute) or equivocal pattern AND high IgG avidity (if performed).

No Evidence of *T. gondii* Infection: IgG-Dye Test Negative and IgM-ELISA Negative

The above diagnostic criteria are routinely used in the daily clinical practice at PAMF-TSL to estimate of the most likely time of the *T. gondii* infection and their performance has been previously validated at the PAMF-TSL (*8–11*).

Data Extraction

For all study family individuals we recorded the date of birth, the unique PAMF-TSL identifying number, the date of specimen collection, the serology and/or PCR results, characterization of the *T. gondii* infection status of those individuals according to the above described diagnostic criteria, any reported clinical manifestations and the risk factors for *T. gondii* infection. Clinical information was limited since it was based on answers to a short questionnaire regarding clinical signs, symptoms and risk factors (eg exposure to cat feces, ingestion of raw/undercooked meat, gardening, none of the above, other), routinely requested to assist in the more accurate interpretation of serologic test results.

Analyses

We calculated the prevalence of group 1 families (primary endpoint) and group 2 families (secondary endpoint) by dividing the number of families in group 1 and 2 respectively, by the total number of study-families over the 20-year study-period.

We used the Kruskal-Wallis non-parametric test to compare the IgG-Dye test titers and IgM-ELISA titers of the index cases across the three family groups. All analyses were done in STATA SE12 (StataCorp LP, College Station, TX, USA).

Supplementary Results

Identification of Eligible Families

Among 97,279 individuals serologically tested for *T. gondii* in the PAMF-TSL database over the 20-year study period, we identified 107 individuals who had in their record the specific code 90, indicating that at least one individual from their household was diagnosed with acute toxoplasma infection and at least one additional household member was serologically tested for *T. gondii* at PAMF-TSL. All samples were sent from diverse laboratories across the United States. Those 107 individuals were grouped in 32 families (Figure 1).

Characteristics of the Study Families

The mean number of additional family members tested per household was 1.7 for families in the first group; 2.6 for families in the second group and 1.3 for families in the third group. In group 1 families, the ICs first tested were three congenitally infected infants, nine pregnant women, one patient with chorioretinitis, four patients with *T. gondii* lymphadenopathy, and one patient with fibromyalgia treated with corticosteroids who had a fatal outcome (Table 1). In group 2 families, the ICs were two *T. gondii*–infected children, two congenitally infected infants, and one patient with chorioretinitis. In group 3 families, the ICs were five pregnant women, one *T. gondii*–infected child, and three congenitally infected infants.

The screening of additional household members of an IC led to the identification of a pregnant woman who had been infected during gestation and was tested because her husband had developed toxoplasmic lymphadenitis (IC 1, group 1) (Table 1). In eight families, the diagnosis of acute toxoplasma infection during pregnancy was made in the mothers after their infants were diagnosed with congenital toxoplasmosis. We also documented one family in which the IC (IC-12, group 1) (Table 1) led to the identification of three additional households being acutely infected. A *T. gondii*–infected meat was considered the most likely source of infection for these four individuals based on the fact that all had eaten raw lamb on a single day at the house of the

IC and also based on the time of onset of their lymphadenopathy and their serological test results.

Serology Titers

In all study families, all additional household members were tested within 1–2 months from the time the family IC was first tested (Table 2). The median IgG-Dye test and IgM-ELISA titers of the ICs in group 1 families were 3072 and 7.9, respectively (Table 2). The corresponding median IgG-Dye test and IgM-ELISA titers for the ICs in group 2 families were 8000 and 7.7 and in group 3 families were 8000 and 4.8, respectively. The IgG-Dye test and IgM-ELISA titers of the ICs were not significantly different across the three family groups ($p=0.27$ and $p=0.07$, respectively).

Risk Factors

For the majority of the study families, the reporting of risk factors for acute *T. gondii* infection was incomplete, precluding a meaningful risk factor analysis between the family groups. In 11/18 families in group 1, in which risk factors were reported for at least one of their household members, exposure to cat feces was reported in 2 families; exposure to food likely contaminated with *T. gondii* was reported in 5 families (e.g., eating raw meat or food handled on surfaces where raw meat was cut and not washed); gardening was reported in 1 family and eating wild game meat was reported in 3 families (the meat was tested at PAMF-TSL and was positive for the presence of *T. gondii* DNA and all individuals who ate this meat were infected) (Table 1). Some families reported more than one risk factor. Two study families reported that they had no known risk factors.

Prevalence of Group-One Families, among Families Tested at PAMF-TSL during 1991–2010 (Primary Endpoint)

The prevalence of group-one families, among the whole cohort of 32 eligible families was 56% (18/32) (Figure 1). In 14 of these group-one families, at least one additional household member had an acute *T. gondii* infection and for the remaining four families, at least one additional household member was found to be recently infected. Specifically, in the family of IC-10 (Table 2) - a pregnant woman with acute toxoplasma infection in the third trimester of her pregnancy- we documented that her 2-year old daughter (Daughter 2) also had a very recently acquired infection (very high IgG and IgA titer, low IgG avidity and acute pattern on the differential agglutination test; although her IgM was negative). In the family of IC-11 (Table

2),an infant with congenital toxoplasmosis, we documented that the child's father had serologic evidence of *T. gondii* infection acquired around the time of his wife's gestation; which was the time during which his wife was also infected (very high IgG titer, low avidity and an acute pattern in the differential agglutination test; although his IgM and IgA were negative). In the family of IC-16 (Table 2), a 70 year old immunocompromised woman with a fatal outcome from disseminated toxoplasmosis, we documented that her daughter also had a recent infection (high IgG titer, positive IgM and IgA and low avidity). In the family of IC-18 (Table 2), a man with toxoplasmic lymphadenopathy, we documented that this man's wife also had a recent infection (high IgG titer, positive IgM and an acute pattern on the differential agglutination test).

References

1. Palo Alto Medical Foundation Toxoplasma Serology Laboratory. http://www.pamf.org

2. Sabin AB, Feldman HA. Dyes as Microchemical Indicators of a New Immunity Phenomenon Affecting a Protozoon Parasite (Toxoplasma). Science. 1948;108:660–3. PubMed http://dx.doi.org/10.1126/science.108.2815.660

3. Naot Y, Remington JS. An enzyme-linked immunosorbent assay for detection of IgM antibodies to Toxoplasma gondii: use for diagnosis of acute acquired toxoplasmosis. J Infect Dis. 1980;142:757–66. PubMed http://dx.doi.org/10.1093/infdis/142.5.757

4. Desmonts G, Naot Y, Remington JS. Immunoglobulin M-immunosorbent agglutination assay for diagnosis of infectious diseases: diagnosis of acute congenital and acquired Toxoplasma infections. J Clin Microbiol. 1981;14:486–91. PubMed

5. Stepick-Biek P, Thulliez P, Araujo FG, Remington JS. IgA antibodies for diagnosis of acute congenital and acquired toxoplasmosis. J Infect Dis. 1990;162:270–3. PubMed http://dx.doi.org/10.1093/infdis/162.1.270

6. Wong SY, Hajdu MP, Ramirez R, Thulliez P, McLeod R, Remington JS. Role of specific immunoglobulin E in diagnosis of acute toxoplasma infection and toxoplasmosis. J Clin Microbiol. 1993;31:2952–9. PubMed

7. Dannemann BR, Vaughan WC, Thulliez P, Remington JS. Differential agglutination test for diagnosis of recently acquired infection with Toxoplasma gondii. J Clin Microbiol. 1990;28:1928–33. PubMed

8. Montoya JG, Huffman HB, Remington JS. Evaluation of the immunoglobulin G avidity test for diagnosis of toxoplasmic lymphadenopathy. J Clin Microbiol. 2004;42:4627–31. PubMed http://dx.doi.org/10.1128/JCM.42.10.4627-4631.2004

9. Montoya JG. Laboratory diagnosis of Toxoplasma gondii infection and toxoplasmosis. J Infect Dis. 2002;185(Suppl 1):S73–82. PubMed http://dx.doi.org/10.1086/338827

10. Montoya JG, Remington JS. Studies on the serodiagnosis of toxoplasmic lymphadenitis. Clin Infect Dis. 1995;20:781–9. PubMed http://dx.doi.org/10.1093/clinids/20.4.781

11. Montoya JG, Berry A, Rosso F, Remington JS. The differential agglutination test as a diagnostic aid in cases of toxoplasmic lymphadenitis. J Clin Microbiol. 2007;45:1463–8. PubMed http://dx.doi.org/10.1128/JCM.01781-06

12. Olariu TR, Remington JS, McLeod R, Alam A, Montoya JG. Severe congenital toxoplasmosis in the United States: clinical and serologic findings in untreated infants. Pediatr Infect Dis J. 2011;30:1056–61. PubMed http://dx.doi.org/10.1097/INF.0b013e3182343096

Technical Appendix Table 1. Serologic test results of family index cases and additionally tested household members in the 5 group-two study families*

IC and additional household members tested	IgG (Dye test)	IgM (ELISA)	IgA (ELISA)	IgE (ELISA)	AC/HS pattern	Avidity	*T. gondii* infection status
IC-19 (child with encephalitis)	4096	4.7	10.5	0.8	Acute	ND	Acute infection
Grandfather	512	0	0.3	0	Equivocal	ND	Chronic infection
Grandmother	<16	0	ND	0	Non acute	ND	No infection
Mother	<16	0	0.2	0	Non acute	ND	No infection
IC-20 (child with JRA)	64000	7.7	8.7	ND	Acute	Low (3.5)	Acute infection
Brother 1	1024	1.1	0.3	ND	ND	High	Chronic infection
Father	128	0.1	ND	ND	ND	ND	Chronic infection
Mother	<16	ND	ND	ND	ND	ND	No infection
Brother 2	<16	ND	ND	ND	ND	ND	No infection
IC-21: Infant with CT	8000	12 (ISAGA)	>11.2	ND	ND	ND	Congenital infection
(Mother)	4096	8.5	2	6	Acute	Low (3.4)	Acute infection
Grandmother	2048	0	ND	ND	ND	ND	Chronic infection
Cousin	<16	ND	ND	ND	ND	ND	No infection
IC-22 (eye disease)	32000	10.7	>24	8.2	Acute	ND	Acute infection
Sister 1	512	0.5	0.6	0.8	Non acute	ND	Chronic infection
Sister 2	<16	0.1	0.2	0	Non-reactive	ND	No infection
IC-23 (Infant with CT)	2048	12 (ISAGA)	13.5	0.5	ND	ND	Congenital infection
(Mother)	4096	2.6	14	0.7	Acute	ND	Acute infection
Brother	512	2.5	1.8	0.3	ND	ND	Chronic infection
Father	<16	1.2	0	ND	ND	ND	No infection

*Mother-infant pairs were counted as 1 unit/household member. Interpretation of results: IgG dye test, positive >16, negative <16; IgM ELISA, positive >2.0, equivocal 1.7–1.9, negative <1.6; IgM ISAGA(for infants <6 mo of age), positive 3–12, negative 0–2; IgA ELISA, positive >2.1, equivocal 1.5–2.0, negative ≤1.4; IgE ELISA, positive >1.9, equivocal 1.5–1.8, negative <1.4; avidity, low <20, equivocal 20–30, high >30. The categorization of AC/HS test results into acute, equivocal, and nonreactive is available at www.pamf.org/serology/images/achs_grid.html. IC, index case-patient; AC/HS, differential agglutination; ND, not done; JRA: juvenile rheumatoid arthritis; ISAGA, immunosorbent agglutination assay; CT, congenital toxoplasmosis. For brother1of IC20 and grandmother of IC21, serologic results could be consistent with chronic infection.

Technical Appendix Table 2. Serologic test results of family index cases and additionally tested household members in the 9 group-three study families*

IC and additional household members tested*	IgG (Dye test)	IgM (ELISA)	IgA (ELISA)	IgE (ELISA)	AC/HS pattern	Avidity	*T. gondii* infection status
IC-24 (Pregnant 32 wks, LN)	8000	8	>11.2	12.7	Acute	Low (1.9)	Acute infection
Husband	<16	0	ND	ND	ND	ND	No infection
IC-25 (Infant with CT†)	8000	0	0.8	ND	ND	ND	Congenital infection
(Mother)	32000	6.7	9.8	2.7	Acute	Low (16.2)	Acute infection
Father	<16	0	ND	ND	ND	ND	No infection
Brother	<16	0.3	ND	ND	ND	ND	No infection
IC-26 (Pregnant 1st trimester)	2048	3	ND	ND	Acute	Low (10.3)	Acute infection
(Baby boy)	1024	0	0.5	ND	ND	ND	Cannot R/O CT
Husband	<16	0	0	ND	ND	ND	No infection
IC-27 (Child-Eye disease)	>16000	1.8	2.5	0.6	Equivocal	ND	Acute infection
Mother	<16	0.2	ND	ND	ND	ND	No infection
Father	<16	0.2	ND	ND	ND	ND	No infection
IC-28 (Infant with CT)	8000	8 (ISAGA)	2.9	ND	ND	ND	Congenital infection
(Mother)	8000	2.7	6.2	ND	Acute	ND	Acute infection
Father	<16	0	0.1	ND	Non-reactive	ND	No infection
IC-29 (Pregnant 1st trimester)	2048	1.9 (3.9 at follow-up)	2.1	ND	Equivocal	ND	Acute infection
(Infant)	32 (at follow-up)	0	0.4	ND	ND	ND	No infection (most likely)
Child	<16	0.5	ND	ND	ND	ND	No infection
IC-30 (Infant with CT)	64000	12 (ISAGA)	>24	12	ND	ND	Congenital infection
(Mother)	32000	7.7	14.2	3.3	Acute	ND	Acute infection
Father	<16	0.1	0.1	0	Non-reactive	ND	No infection
IC-31 (Pregnant, fever)	1024	5.6	6.9	3.1	Acute	ND	Acute infection
(Baby girl)	16 (at 6.5 mo)	0	0.6	ND	ND	ND	No infection (most likely)
Husband	<16	0	ND	ND	ND	ND	No infection
IC-32 (Pregnant 2nd trimester)	8000	5.8	ND	ND	Equivocal	Low (8.6)	Acute infection
Household member 1	<16	0.5	ND	ND	ND	ND	No infection
Household member 2	<16	0.5	ND	ND	ND	ND	No infection

*Mother-infant pairs were counted as 1 unit/household member. Interpretation of results: IgG dye test, positive ≥16, negative <16; IgM ELISA, positive ≥2.0, equivocal 1.7–1.9, negative ≤1.6; IgM ISAGA(for infants <6 mo of age), positive 3–12, negative 0–2; IgA ELISA, positive ≥2.1, equivocal 1.5–2.0, negative ≤1.4; IgE ELISA, positive ≥1.9, equivocal 1.5–1.8, negative ≤1.4; avidity, low ≤20, equivocal 20–30, high ≥30. The categorization of AC/HS test results into acute, equivocal, and nonreactive is available at www.pamf.org/serology/images/achs_grid.html. IC, index case-patient; AC/HS, differential agglutination; LN, lymphadenopathy; CT, congenital toxoplasmosis; ND, not done; ISAGA, immunosorbent agglutination assay. Serologic results were consistent with acute infection, despite equivocal AC/HS, in IC29, IC32.
†Infant with congenital toxoplasmosis with hydrocephalus and hepatosplenomegaly.
